# Evaluation of a multi-site health services psychology training program for telehealth and integrated behavioral health

**DOI:** 10.3389/fpsyg.2024.1339319

**Published:** 2024-03-13

**Authors:** Carly McCord, Whitney Garney, Kristen Garcia, Blanca Macareno, Meredith Williamson

**Affiliations:** ^1^Texas A&M Telehealth Institute, Texas A&M University Health Science Center, Bryan, TX, United States; ^2^Department of Educational Psychology, School of Education and Human Development, Texas A&M University, College Station, TX, United States; ^3^Department of Psychiatry and Behavioral Sciences, School of Medicine, Texas A&M University Health Science Center, Bryan, TX, United States; ^4^Department of Health Behavior, School of Public Health, Texas A&M University Health Science Center, Bryan, TX, United States; ^5^Department of Primary Care and Rural Medicine, School of Medicine, Texas A&M University Health Science Center, Bryan, TX, United States

**Keywords:** telehealth, integrated behavioral health, health services psychology, training, interdisciplinary care, medically underserved, rural

## Abstract

**Introduction:**

Training future providers in telehealth and integrated care models can improve access and outcomes, especially among rural and underserved populations. The (blinded) project implemented behavioral health training for health service psychology doctoral students with three partner organizations. Trainees received both experiential and didactic training in telehealth and integrated behavioral health. Telehealth was utilized for remote warm hand-offs, hybrid shared appointments, therapy sessions, coordination with providers, and supervision. Program elements included opportunities for consultations with experts in other disciplines, supportive mentorship, exposure to various parts of a healthcare system, and interactions with diverse clients.

**Methods:**

The (blinded) training program evaluated trainee outcomes using fourteen interviews and three focus groups. Interviews and focus groups examined aspects of the program that contributed to trainees’ knowledge, skills, and attitudes.

**Results:**

Evaluation results revealed increased levels of trainee confidence, autonomy and independence. Training reportedly enabled improved ability to collaborate and communicate with other professions, increased flexibility and adaptability, and openness to others’ ideas. Trainees reported the program’s use of telehealth enhanced awareness of their own skills and team members’ perspectives of technology in care delivery.

**Discussion:**

Descriptions of the three care models, lessons learned, and qualitative results about trainee outcomes can be translated into best practices for workforce development and enhance psychology trainees’ self-awareness and ability to incorporate others’ viewpoints about technology and treatment approaches into healthcare.

## 1 Introduction

Currently, two undeniable zeitgeists in United States (US) healthcare are the integration of mental and physical health in US healthcare systems and the integration of technology in health services. Both trends have well documented evidence for improvements in accessibility, availability and quality (i.e., improved health outcomes) (Funk et al., 2008; Segal et al., 2022; Shah et al., 2022). With this evolution of US health systems, training for future and current health professionals must be updated to provide education and practice for relevant knowledge, skills, and attitudes regarding provision of services via telehealth (McCord et al., 2015; Perle, 2021), as well as providing interdisciplinary and integrated care (Blount and Miller, 2009; Bluestein and Cubic, 2009; O’Donohue et al., 2009; Hall et al., 2015).

The terms “interdisciplinary” and “integrated” are often used inconsistently amongst professionals and in the literature since the terms are not mutually exclusive or mutually exhaustive on their own. Integrated care is, by definition, interdisciplinary, but interdisciplinary care does not necessarily imply behavioral health and physical healthcare is integrated into the same setting. Interprofessional and interdisciplinary are also used inconsistently. Boon et al. (2009) makes the distinction that interdisciplinary care is “a model of professions working closely together (i.e., collaborating) in the delivery of care.” Whereas, “integrated” care is “subsumed into a single organizational framework (i.e., integration)” (Boon et al., 2009). The project described here is interdisciplinary and the training sites all have some level of integration of behavioral health services with primary care services offered within a US health system.

The term telehealth is also used to describe many ways technology is applied to health to foster connections from a distance. As stated by the Center for Connected Health Policy, “There is no single definition for telehealth” (CCHP, 2023). And “telehealth is a broad term that encompasses a variety of telecommunications technologies and tactics to provide health services from a distance. Telehealth is not a specific clinical service, but rather a collection of means to enhance care and education delivery” (CCHP, 2023). In this case, telehealth is used both for clinical and educational purposes.

### 1.1 Telehealth training and competencies

Telehealth training for US providers was limited and housed within specialized programs for many years (Papanagnou et al., 2015). However, with the transition to telehealth as a common mode of healthcare service delivery during COVID-19, a need for expanded training emerged. Training in telehealth can reduce or eliminate the perceived barriers of using telehealth resulting in higher levels of provider comfort and satisfaction with delivering services through telehealth (Traube et al., 2021). Some examples of telehealth competencies for providers across various disciplines have been proposed over the years. One example of telepsychology competencies covers seven domains: (1) clinical evaluation and care, (2) virtual environment and telepresence, (3) technology, (4) legal and regulatory issues, (5) evidence based and ethical practice, (6) mobile health technologies, and (7) telepractice development (Hertlein et al., 2021). The American Academy of Medical Colleges also published telehealth competencies for licensed medical professionals that addressed the following domains: (1) patient safety and appropriate use of telehealth, (2) access and equity in telehealth, (3) communication via telehealth, (4) data collection and assessment via telehealth, (5) technology for telehealth, and (6) ethical practices and legal requirements for telehealth (Association of American Medical Colleges, 2021). Additionally, the American Medical Association offers training for physicians regarding procedures and ethics (Telehealth Training, 2023). Nursing, social work, and other disciplines have also published on the need for telehealth training, but there is little documented regarding the actual curricula and trainings for professionals and the utilization of telehealth within integrated care.

### 1.2 Integrated behavioral health in primary care

The Primary Care Behavioral Health Model (PCBH) is a population health model of behavioral healthcare that is designed to increase access and decrease stigma for behavioral health services by providing them within primary care settings. The integrated PCBH team includes a licensed behavioral health professional who functions as a Behavioral Health Consultant (BHC) and shares responsibility of providing direct patient care with the primary care team (American Psychological Association, 2022). The BHC’s role is implementing prevention, early identification, and intervention strategies across the clinic in addition to treatment provision for physical and behavioral health concerns across the lifespan (American Psychological Association, 2022). Amongst ethnically and racially diverse patient populations along with low-income and other underserved populations, health outcomes have shown improvements when they are able to access behavioral health within primary care settings (American Psychological Association, 2022). Despite research support for integration of behavioral health into primary care settings, there is a paucity of behavioral health training programs preparing the behavioral health workforce to provide interdisciplinary care in integrated settings. There is a critical need to address the behavioral health workforce shortage, especially evident in rural communities, where primary care providers have assumed the role as providers of behavioral health services. In response, behavioral health services are becoming more widely implemented within primary care settings. However, the opportunity to provide education and training in the primary care and behavioral health integrated care model is limited (Blount and Miller, 2009; Bluestein and Cubic, 2009; O’Donohue et al., 2009; Hall et al., 2015). Therefore, adapting both the education and training models within psychology enables psychologists to become full partners in healthcare rather than silo mental health specialists to better serve those in need (Cubic et al., 2012).

### 1.3 Graduate Psychology Education Program and the (blinded) project

In response to a recognized need for increased public health resources the US Department of Health and Human Services (HHS) developed a five-point strategy that included access to better prevention, treatment, and recovery services in interdisciplinary and primary care settings. Additionally, HHS aimed to improve behavioral health workforce programs through existing mechanisms such as the Graduate Psychology Education Program (GPE) (Health Resources & Services Administration, 2020). This GPE program emphasized the inclusion of interdisciplinary teams, integrated behavioral health (IBH) models in primary care, and the use of telehealth for service delivery. In response, the (blinded) university was awarded a 3 year plus no-cost extension year grant project.

This (blinded) project provided experiential training opportunities (refer to section “2.1 Training sites”) for health service psychology doctoral trainees [i.e., trainee(s)] with three partner clinical entities in the US. All trainees received both experiential and didactic training in telehealth and integrated behavioral health. Training models included telehealth and in-person encounters with telehealth being utilized in a variety of ways. For example, telehealth was used for remote behavioral health services on the same day as their primary care visit, hybrid interdisciplinary appointments, and virtual psychotherapy sessions. Program elements included opportunities for consultations with experts in other disciplines, supportive mentorship, exposure to various parts of a healthcare system, and interactions with diverse clients. Two of the experiential practicum sites were clinical entities within an academic health science center. The third was a federally designated Rural Emergency Hospital with associated Rural Health Clinics.

An evaluation team, external to project implementation, was engaged. The evaluation focused on the overall process of implementation and perceptions of the training, in addition to tracking trainee demographics and performance measures required by the funder. Through qualitative assessments, evaluators aimed to gain contextual insights on the perceptions of working in interdisciplinary teams, reflections (positive and negative) on the experience, and logistical aspects. Qualitative assessments were targeted to explore the follow evaluation question:

Evaluation question: To what extent was a comprehensive training program implemented to include IBH, and other specialized training to work with rural and underserved populations?

## 2 Materials and methods

The evaluation team conducted interviews and focus groups with health service psychology doctoral trainees participating in the (blinded) project to assess the overall training experience after all trainees received both experiential and didactic training across cohorts. To designate the level of integration of each practicum site, the project staff used the Standard Framework for Levels of Integrated Healthcare, as described below when introducing each practicum site. This tool was used for informational purposes in preparing for work in each setting but not included in the project evaluation (SAMHSA-HRSA Center for Integrated Health Solutions, 2019). The framework has six levels ranging from minimal collaboration to full collaboration in a transformed/merged integrated practice.

### 2.1 Training sites

All trainees received a standard set of trainings, including a telehealth component, which provided a common baseline for the experience. However, the combinations of different health disciplines and level of integration in care varied by site. Telehealth utilization rates and strategies also differed by site. The (blinded) project partnered with each of the training sites (i.e., Site One, Site Two, and Site Three) and had a signed Memorandum of Understanding (MOU) before beginning services. Health service psychology doctoral trainees received stipends through the project to provide services as part of their training program (not internship) experience. Trainees were all expected to complete 1 year of training at their assigned site. Site One had previously trained a few doctoral students, but none that were associated with this expanded project, Site Two had previous training for social work fellows but was a new site for psychology doctoral students, and Site Three was a new site established for this project. Across sites, trainees spent (on average) 10–12 h per week providing direct clinical services and spent the remaining time in case management (5 h), case consultation (2 h) individual supervision (1 h) and training (2 h) for a total of 20 h commitment weekly. Broadly, trainees received experience in the following categories recognized by the Health Resources and Services Administration (HRSA) as priorities: telehealth, primary care, medically underserved community, and/or rural area.

#### 2.1.1 Site One

Site One was a large primary care clinic that is part of an academic health science center and houses a family medicine residency program. Site One is at Level 6 of integration with full collaboration in a transformed/merged integrated practice. Site One includes a well-established integrated behavioral health program run by a licensed psychologist utilizing the PCBH model. Interdisciplinary team-based consultation services with both medical and behavioral health providers are also a part of the integrated behavioral health program. Synchronous videoconference and audio-only telehealth were utilized as needed (based on patient, provider, and pandemic needs) in all integrated and interdisciplinary services.

#### 2.1.2 Site Two

Site Two was also part of the academic health science center from Site One and offers comprehensive psychiatric services, including diagnostic evaluation and assessment, group and individual psychotherapy, and medication management. Site Two met criteria for Level 3: basic collaboration onsite for sites that are co-located with primary care. The site offers specialized treatment of bipolar disorders, depression disorders, anxiety disorders, substance use disorders, psychosis, as well as geriatric and medical-psychiatric conditions. Site Two provides training for psychiatry students, social work postgraduate fellows, and psychiatry residents. Site Two utilized synchronous videoconference and audio only telehealth options for intake and follow-up therapy appointments and for care coordination calls with other providers.

#### 2.1.3 Site Three

Site Three was a safety net, critical access health system that has a federal designation as a Rural Emergency Hospital with three associated, federally designated Rural Health Clinics (RHCs). The system provides emergency services, primary care, chiropractic, psychology, podiatry, among other services. As a part of the project, trainees participated in an entirely remote integrated model and provided telebehavioral health services to the three RHC’s and were integrated into a shared electronic medical record (EMR) used by the entire system. This project was the system’s first attempt at integrating behavioral health into the workflow other than referrals to an existing part-time psychologist, and the model of care designed for this site met criteria for Level 4: close collaboration onsite with some onsite integration. Site Three exclusively used telehealth services provided via synchronous videoconference and audio only options for individual and group therapy and were available for warm hand-offs from medical providers. While virtual warm hand-offs were available, there was limited use at this site.

### 2.2 Sample

All health service psychology doctoral trainees (Table 4) placed at the training sites (refer to section “2.1 Training sites”) participated in the (blinded) project and were invited to participate in focus groups (years 1, 3, and 4) and interviews (years 1 and 2). Evaluators were familiar with trainees but did not have an existing relationship with the trainees outside of their role in project evaluation. Trainees were recruited by email for both interviews and focus groups. An information sheet regarding each research study component was emailed to participants to review and sign, prior to participation. Participants were able to email the completed consent form to evaluators or return the completed consent form in person. The total number of trainees in each cohort, as well as the numbers of trainees who participated in each data collection activity is listed by cohort in Table 1. All participants consented to evaluators recording the interviews and focus groups.

**TABLE 1:** Number of trainees, separated by data collection method.

Cohort	Total trainees	Total focus group participants	Total interview participants
1 (2019–2020)	8	2 (25%)	7 (87.5%)
2 (2020–2021)	8	–	7 (87.5%)
3 (2021–2022)	8	2 (25%)	–
4 (2022–2023) (No cost extension project year)	4	3 (75%)	–

### 2.3 Data collection

Qualitative data was collected through interviews and focus groups to gain contextual insights into the trainee experiences. The interviews and focus groups were exploratory in nature and intended to gain feedback on broad programmatic aspects. They were conducted separately to elicit feedback on diverse topics. Topics in focus groups regarded interdisciplinary training and were determined to potentially benefit from group interactions in which one participant’s answer can spur responses from other participants. Topics discussed in interviews focused on individual experiences and intentions for future use. All focus groups and interviews were conducted by a member of the evaluation team with master’s level training in public health or health education, including research methods. The (blinded) Institutional Review Board approved the protocol and materials.

#### 2.3.1 Focus group

Focus group scripts included questions regarding the training received. The focus group script included 9 questions and lasted approximately 45–60 min (Table 2). Example questions included “What were you able to learn with, from, and about interprofessional team members to enhance care?” and “Describe your ability to utilize an interprofessional team approach with the patient to assess the health situation and provide whole person care?”. Focus groups were conducted virtually through Zoom in cohort 1 (due to safety considerations regarding COVID-19) and in person for cohorts 3 and 4. A focus group was not conducted with cohort 2 due to reprioritization of qualitative data collection to reduce participant burden and the difficulty convening participant groups following COVID-19. With the permission of the participants, the focus groups were recorded, and transcribed by an external transcription firm with a signed confidentiality agreement. Focus group facilitators took notes with a standard note taking template to supplement the audio recording. Questions were not provided to the participants prior to the focus groups.

**TABLE 2:** Focus group script.

Question number	Question
1	Describe your interprofessional experience while participating in the (blinded) program.
2	Describe how you communicated and shared information with other team members outside of your profession. *Probe: Did you use terminology that all members of the team could understand?*
3	How did your role and responsibilities contribute to the interprofessional team you worked with? *Probe: Were roles adequately overlapped among team members?* *How did others’ skills and knowledge complement the overall team?*
4	What were you able to learn with, from, and about interprofessional team members to enhance care? *Probe: What abilities and contributions did the interprofessional team members make to patient care?*
5	How was conflict handled among team members? *Probe: What steps were taken for conflict resolution?*
6	Describe your ability to utilize an interprofessional team approach with the patient to assess the health situation and provide whole person care?
7	What roles do the patient and their family play in decision-making in interprofessional care?
8	How will you apply the skills you learned from your interprofessional experience to your future practice? Probe: How does the interprofessional experience relate to your long-term career goals?
9	Is there any other information you would like to share about your interprofessional experience?

#### 2.3.2 Interviews

Evaluators developed interview scripts with questions designed to learn more about the trainee’s experience with the project, including positive and negative aspects, as well as intentions to utilize the skills in the trainee’s future career. The interview script included 15 questions and lasted approximately 30 min (Table 3). Example questions included “How did (blinded) increase your ability to work with rural and/or underserved populations?” and “In what ways did the (blinded) program change the way you will practice psychology?”. Interviews were conducted over Zoom with an audio only connection, by a member of the evaluation team with master’s level training in public health or health education, including research methods. With the permission of each interviewee, all interviews were recorded, and transcribed by an external transcription firm with a signed confidentiality agreement. Each call was conducted by one interviewer with one participant at a time. Questions were not provided to the interviewees prior to the calls.

**TABLE 3:** Interview script.

Question number	Question
1	Describe the (blinded) program in your own words.
2	Please describe some key learning experiences you had through this practicum.
3	How were you able to apply (blinded) content trainings to your experiential training? *Probe: How were you able to apply the knowledge and skills gained through trainings on substance use disorder (SUD)/OUD to practice?*
4	How did (blinded) increase your ability to work with rural and/or underserved populations?
5	How did (blinded) increase your access to resources to practice psychology and serve rural and/or underserved populations?
6	What additional resources do you believe would be useful for future practicum students?
7	What was the most valuable aspect of this practicum opportunity?
8	What was the least valuable aspect of this practicum opportunity?
9	What changes would you recommend to improve the (blinded) program?
10	In what ways did the (blinded) program change the way you will practice psychology?
11	To what extent do you feel you will be utilizing the knowledge and skills you gained to your future practice?
12	What skills did you acquire through this program that you will be able to highlight in your resume, cover letters, and/or interviews?
13	How does this practicum opportunity relate to your long-term career goals?
14	What are your post-graduation intentions?
15	Is there any other information you would like to share about your experience with (blinded)?

### 2.4 Data analysis

Following each cohort of data collection, two trained members of the evaluation team conducted a thematic analysis using an open coding scheme. Evaluators followed established qualitative analysis processes that began with reviewing the text and identifying important data elements throughout the transcripts (Lincoln and Guba, 1985; Attride-Stirling, 2001). For interviews, coded segments were integrated to establish emergent themes that occurred across interviews within each cohort. For focus groups, coders identified key segments that occurred throughout the sessions.

Following independent analysis, the coders met to compare coded segments and discuss categorization. Discrepancies in thematic coding and categorization were resolved through group consensus before combining the results into an overall summary. Multiple members of the evaluation team with varying levels of program knowledge coded and themed the transcripts. Both interviews and focus groups were first analyzed by cohort and then later combined across cohorts for overall themes that persisted throughout the project.

## 3 Results

Table 4 presents trainees’ demographics by cohort year. Over the 4 year project period, the trainees were majority female (*n* = 24, 86%), not Hispanic/Latinx (*n* = 20, 71%), White (*n* = 21, 75%), did not have a rural residential background (*n* = 26, 93%), did not have a disadvantaged background (*n* = 23, 82%), enrolled in the counseling psychology (*n* = 19, 68%) APA-accredited doctoral program, and in their 4th academic year (*n* = 11, 39%).

**TABLE 4:** Demographics of trainees by cohort year.

	Cohort 1 (2019–2020)	Cohort 2 (2020–2021)	Cohort 3 (2021–2022)	Cohort 4 (2022–2023)
**Total**				
	8	8	8	4
**Gender**				
Male	1 (12.5%)	1 (12.5%)	2 (25%)	0 (0%)
Female	7 (87.5%)	7 (87.5%)	6 (75%)	4 (100%)
**Ethnicity**				
Hispanic/Latinx	2 (25%)	1 (12.5%)	3 (37.5%)	2 (50%)
Not Hispanic/Latinx	6 (75%)	7 (87.5%)	5 (62.5%)	2 (50%)
**Race**				
Asian	2 (25%)	1 (12.5%)	0 (0%)	0 (0%)
Black/African American	1 (12.5%)	0 (0%)	2 (25%)	0 (0%)
White	5 (62.5%)	6 (75%)	6 (75%)	4 (100%)
Biracial	0 (0%)	1 (12.5%)	0 (0%)	0 (0%)
**Rural background**				
Yes	0 (0%)	1 (12.5%)	1 (12.5%)	0 (0%)
No	8 (100%)	7 (87.5%)	7 (87.5%)	4 (100%)
**Disadvantaged background**				
Yes	1 (12.5%)	0 (0%)	4 (0.5%)	0 (0%)
No	7 (87.5%)	8 (100%)	4 (0.5%)	4 (100%)
**Primary discipline**				
Counseling psychology	5 (62.5%)	6 (75%)	5 (62.5%)	3 (75%)
School psychology	2 (25%)	1 (12.5%)	2 (25%)	0 (0%)
Clinical psychology	1 (12.5%)	1 (12.5%)	1 (12.5%)	1 (25%)
**Academic year**				
2	1 (12.5%)	2 (25%)	1 (12.5%)	2 (50%)
3	0 (0%)	4 (50%)	4 (50%)	1 (25%)
4	5 (62.5%)	2 (25%)	3 (37.5%)	1 (25%)
5	2 (25%)	0 (0%)	0 (0%)	0 (0%)

### 3.1 Participant training

Table 5 presents the various didactic topics and descriptions that all the trainees received through either synchronous (e.g., in-person or hybrid) or asynchronous format.

**TABLE 5:** Didactic training topics and descriptions.

Topic	Description/key topics
**Telehealth core**	
Telehealth 101	• History of telehealth • Telehealth terminology and modalities • Telehealth pros and cons • Introductory telehealth literature review
Multicultural aspects of rural health	• Helps clinicians identify barriers to healthcare in rural populations • Explores ways to approach rural health compared to healthcare in an urban area
Laws and ethics of telehealth	• Reviews legal and ethical concerns in telehealth • Reviews the American Psychological Association’s (APA) guidelines for the practice of telepsychology • Reviews implications of PSYPACT for the field of telebehavioral health
Managing crises and emergencies in telehealth	• Reviews risk trends and risk assessment • Reviews developing written plans for crises and emergencies and effective safety planning
Treatment considerations	• Reviews unique elements of telehealth practice and how to overcome potential challenges • Expanded telehealth literature review
**Integrated behavioral health (IBH) core**	
Introduction to integrated behavioral health	• Provides strategies to intervene as an IBH team • Reviews what is health psychology, the biopsychosocial model, phases of a 30-min warm handoff appointment, very brief universal interventions (30 s to 2 min), quick therapy interventions (i.e., psychoeducation, behavioral activation, cognitive restructuring, etc.), ethical considerations, and resources for learning more • Practice role plays of how to interact with health professionals from other disciplines as part of clinical care
**Opioid use and substance use disorder core**	
Introduction to medication assisted treatment	• Discuss “Harm Reduction” as a patient outcomes and public health strategy • Describe the risk factors and clinical features of opioid use disorder (OUD) • Describe the Food and Drug Administration approved treatment modalities for OUD, and differentiate between treatment strategies
Opioid overdose education and naloxone administration (OENA)	• Reviews opioid definitions, scope and magnitude of current problem, risk of opioid use/misuse, signs/symptoms of opioid overdose, harm reduction strategies designed to engage communities and include at-risk and underserved populations in the fight against the opioid crisis • Describes the specific steps required to reverse an overdose using naloxone and ensure that a potential overdose victim is connected to the next level of emergency care • Trainees receive a naloxone overdose reversal kit thanks to funding from the state of Texas granted to the University of Texas San Antonio Texas Targeted Opioid Response Team
**Specialized core**	
Cognitive-behavioral therapy for chronic pain (CBT-CP)	• Group therapy: recruitment and retention, process/dynamics, cohesion, confidentiality, and multicultural considerations • Conducting groups via telehealth • Introduces trainees to the manual, the importance of fidelity, and steps to prepare to deliver the group intervention
Screening, brief intervention, and referral to treatment (SBIRT)	• Overview of the SBIRT model • Reviews motivational interviewing • Resources for SBIRT and opioid use disorder
Social determinants of health (SDOH)	• Understand the social determinants of health factors and use this data in diagnosis and treatment planning
How to apply for a national provider identifier (NPI) number	• Step-by-step guide on how to apply for a NPI number

Through interviews and focus groups, trainees highlighted a variety of learning experiences. The key themes are detailed below. Coding resulted in main themes regarding telehealth experiences, interdisciplinary collaboration, and supervision, with associated subthemes.

### 3.2 Telehealth experiences

Trainees received valuable training experience through both telehealth and in person modalities and highlighted the importance of experience working in a hybrid, flexible team. Trainees highlighted the value of telehealth in reaching rural and underserved communities. One participant stated “I think that all of those experiences really added to my resources and knowledge and skill and ability to serve clients from different socioeconomic groups, different rural areas, or marginalized individuals.” (interview participant D: cohort 1). Overall, they felt that skills in providing telehealth consultations, in partnership with an interdisciplinary team would be utilized in future professional employment.

Trainees gained experience in remote, synchronous communication with patients and the provider care team (e.g., therapy conducted by videoconference and phone), but the telehealth training went beyond this aspect. Using a broad definition of telehealth to include digital communication both synchronous and asynchronous, trainees reported that they gained experience in communicating with other providers virtually to coordinate, give referrals, and communicate about common patients. This included real time communication for warm handoffs, as well as communication through the EMR, and secure emails and chat functions.

### 3.3 Interdisciplinary collaboration

Experience working in an interdisciplinary team was a key theme for trainees. They identified a variety of learning experiences and had a high level of appreciation for the necessity of diverse perspectives in providing holistic patient care through interdisciplinary collaboration.

#### 3.3.1 Improved client care

Trainees felt that interdisciplinary care resulted in provision of high quality, client-centered care. Providers were able to learn from other team members and other disciplines represented on the interdisciplinary team through working together to develop the patient care plans. “I think in every patient encounter, there was always the opportunity to utilize the whole team and everybody’s different expertise and viewpoints to care for the one patient.” (focus group: cohort 1). Through this collaboration, providers were able to communicate and “negotiate” the care plan to provide complementary care and address the holistic needs of each patient, rather than addressing individual health concerns through siloed treatment. One trainee stated, “I’ve been interested in community mental health. I think this has helped me just operationalize that idea in a sense. Being able to see like this is what it looks like to start something up or to collaborate with a different team of people, a different setting, different treatment modalities, etc.” (interview participant B: cohort 2).

Additionally, addressing the patient holistically, including diverse provider perspectives, empowered the patients to be involved in their own healthcare decision making because the presence of a psychologist “… can be a really good bridge or a new experience for patients, where they may feel like they have more autonomy and choice in what their treatment looks like.” (focus group: cohort 1). For example, in a traditional provider-patient relationship, the conversation can be one directional, but including behavioral health and social work as part of the standard care allowed for patients to create a dialog and play an active role in their healthcare decision making. The unique integration of perspectives also included more opportunity for involvement of the family and/or support system in patient care.

#### 3.3.2 Applying models of care

The concept of providing integrated care was highlighted as being directly applicable to trainees’ future careers. One trainee stated, “After being in this. training opportunity, it’s hard to imagine, like being somewhere where I’m not. plugged in with other professionals in different fields” [focus group: cohort no cost extension (NCE)]. This was reiterated multiple times with another participant stating “I think it really motivated me to want to work in an integrated care setting and specifically in primary care. I feel like it changed my whole career trajectory in a lot of ways” (interview participant A: cohort 2).

Additionally, with the limited length of appointment time for treatment using the integrated care model as opposed to traditional standalone behavioral health appointments, trainees had the opportunity to practice brief intervention models to maximize the time available with patients. “I think it’s a big help. For patients to be able to take care of all of their needs in one appointment I think is really helpful” (focus group: cohort 1).

#### 3.3.3 Professional skills

Improved professional skills that would be applicable to a broad range of professional environments were gained through this experience. Trainees gained experience in both in-person and telehealth modalities for working in an interdisciplinary care team. They highlighted practice in effective communication, including important skills such as how to explain diagnoses to patients and other professionals, and how to communicate among interdisciplinary teams to come to a consensus among providers and provide high quality patient care. One participant stated “If we’re using a term that they don’t understand, making sure we explain it, not just throwing that term and assuming they will understand” (focus group: cohort 3).

Working on an interdisciplinary team rather than providing solo care also gave the opportunity to practice flexibility—“We’re kind of presenting our conceptualizations, which is from a very different background than the, the [family medicine] residents that we’re working with, they obviously see it very differently and we all kind of have shared goals and that we want to see the patient better, in a better spot, but I think we all go about it differently.” (focus group: cohort 4). Establishing a common language and learning to work with different disciplines who have diverse skill sets and expertise requires flexibility in how to approach patient care.

#### 3.3.4 Increased confidence

Training in an interdisciplinary setting provided the opportunity to develop skills that would not be available in traditional settings. Trainees got the opportunity to gain confidence in conveying their knowledge and expertise in working with other professionals as well as practice in resolving professional disagreements such as different opinions on diagnosis or treatment. “It’s like really easy to question your competencies about what you’re doing. And I think this really kind of taught that we do have expertise in areas and we really can kind of teach other professionals to get a more holistic team together to care for someone holistically from a whole perspective, like physically and mentally” (focus group: cohort 4).

### 3.4 Supervision

One of the key factors highlighted by trainees was the role of the supervisor. Trainees indicated that supervisors modeled constructive interactions with other members of the interdisciplinary team. Additionally, the supervisors challenged and empowered the students to not only provide high quality patient care, but to also have the confidence to participate as an integral member with unique expertise, but complementary expertise to the other professionals on the care team. Collaborative interactions and communication were modeled among supervisors. “[supervisor] usually doesn’t go into the room with the client so she kind of facilitates the discussion on what makes you think it’s the diagnosis and what makes you think it’s this versus that, so that both of us can think out loud through it” (focus group: cohort 3).

#### 3.4.1 Program barriers and potential improvements

When asked about the least useful components of the training program and recommendations for improvement, interview responses in both cohorts 1 and 2 focused on logistical issues associated with technology, organizational logistics of working with newly established partner organizations, and transparency of assignment to training locations. Additionally, participants mentioned improving the role of didactic trainings, but responses were mixed as to whether the program should include more or less didactic training. One highlighted dichotomy with didactic trainings was that participants expressed an appreciation for learning about a variety of topics but felt they had limited opportunity to apply some skills with their patient population.

Noticeably absent from the responses were negative reflections on working within interdisciplinary teams. On the contrary, participants felt that conflicts or disagreement on client care decisions were handled with respect and approached from a learning perspective, “I didn’t really come across conflict a lot, honestly.just because everyone was open to learning both from us and them and understanding that like they bring in different resources and understanding” (focus group: cohort 4).

## 4 Discussion

This paper described a multi-site project that provided health service psychology doctoral students with training in telehealth, integrated care models, and substance use disorder treatment. The project demonstrated that interdisciplinary care is a valuable enhancement to patient care with the potential to evolve quality training for the next generation of health professionals.

### 4.1 Limitations

This evaluation is limited to a single training program with three sites. A comprehensive evaluation of HRSA GPE projects or other programs that incorporate both telehealth and IBH training would provide more generalizable themes and insights. As mentioned in the introduction, the terms interdisciplinary (or interprofessional) and integrated are often used interchangeably by professionals and within the literature. The same was found to be true when coding trainee responses that seemed to use interdisciplinary, interprofessional, and integrated interchangeably. This may represent a key learning opportunity for future research and for training programs to ensure learners understand the difference and advance specificity in this language as opposed to promulgating continued ambiguity of these terms.

Another limitation is the structure of how data collection was integrated into the training program. While the evaluators were external to the evaluation team and efforts were made to make it explicitly clear that participation was optional and feedback would remain confidential, it is possible that participants felt compelled to participate and also to respond positively about the program. The originally planned data collection methods utilized both interviews and focus groups. However, this was not feasible given the trainee burden and constraints. Therefore, data collection varied across years resulting in some inability to review the same data points across all 4 years. Focus groups included less participants than ideal as scheduling was confounded by the impacts of COVID-19, especially in scheduling focus groups for cohorts 1 through 3. The cohort 2 focus group was skipped altogether due to in person scheduling concerns and difficulty of scheduling a virtual focus group amidst COVID-19. Therefore, feedback may have been limited to certain perspectives. With only 2 participants each, the cohort 1 and 3 focus groups should be considered dyadic interviews, although intended as focus groups.

### 4.2 Lessons learned

#### 4.2.1 Telehealth

For decades, telehealth research has focused on methods of non-inferiority to demonstrate that remote care is equivalent to in-person care and has consistently found that telehealth is as good as or better than in-person care on a variety of metrics (Langarizadeh et al., 2017; Lin et al., 2022). This consistency has given a solid foundation for innovation and application of telehealth in real world-settings, like was done in this project. This strong platform gives way for continued evolution in research and practice to better understand the nuances needed to match telehealth solutions with organizational capacity and patient needs. Across sites, it was evident that while technology had the ability to solve challenges in care, implementation challenges and provider readiness were barriers to fulfilling the potential available through the adoption of telehealth workflows and technology. The health service psychology doctoral trainees in the project were primed and seemingly excited to incorporate telehealth, whereas the sites demonstrated greater difficulty with implementation. The emphasis on telehealth training in the call for proposal from the HRSA GPE program predated the COVID-19 pandemic, representing the mounting urgency to include telehealth experiences in training. Although the pandemic placed necessary pressures on systems to use telehealth for service delivery, training sites still experienced a variety of challenges in adoption and implementation.

#### 4.2.2 Integrated behavioral health

By providing integrated care experiential training to three unique sites, trainees were exposed to different challenges and successes within their placements that were shared during project group meetings and informally as trainees discussed their sites with peers. Regardless of site assignment, trainees saw the value and importance of caring for the whole patient and of using technology to enhance care. This key learning was driven both from the things they saw going well at their site in addition to the things that weren’t functioning as optimally as they hoped. Trainees were taught about the levels of integration in didactics and likely set their hopes on Level 6, full collaboration in a transformed/merged integrated practice. The experiential training exposed them to the real-life challenges of systems working toward this goal while simultaneously incorporating telehealth and technology options to enhance care, coordination, and training.

### 4.3 Future directions

There is a parallel process occurring for telehealth and integrated behavioral health training as it relates to the development of competencies (McDaniel et al., 2014; Kinman et al., 2015). In both cases, there are limited guidelines or established models for competency development. When present, guidance does not typically extend across professions or level of professional development (i.e., trainees vs. licensed professionals). Establishing competencies in these practice areas would require collaboration between many accrediting bodies and should consult a diverse set of stakeholders including (but not limited to) patients, providers, educators, scholars, and professional associations. Projects such as this one that represent real-world applications of these models of care can inform relevant stakeholders in this process. With an aim of building to the development of competencies, similar evaluations should be conducted with other professionals included on interdisciplinary teams (physicians, social workers, etc.), as well as interdisciplinary teams in other settings.

## 5 Conclusion

On their own, telehealth and integrated care models continue to be ripe for innovation in application in practice and for evaluation and research. Organizations, providers, and training programs should strive to incorporate both in care models since the documented benefits in availability, accessibility, and quality of care continue to mount. Moreover, telehealth and integrated care models should not be considered separately. The incorporation of the intersection of these models, as described in this project and subsequent evaluation, represents the future of health services training and progression from non-inferiority research of these models.

## Data availability statement

The raw data supporting the conclusions of this article will be made available by the authors, without undue reservation.

## Ethics statement

The studies involving humans were approved by the Texas A&M University Institutional Review Board. The studies were conducted in accordance with the local legislation and institutional requirements. The participants provided their written informed consent to participate in this study.

## Author contributions

CM: Conceptualization, Funding acquisition, Project administration, Supervision, Writing – original draft, Writing – review & editing. WG: Conceptualization, Funding acquisition, Supervision, Writing – review & editing. KG: Data curation, Formal Analysis, Writing – original draft, Writing – review & editing. BM: Project administration, Writing – original draft, Writing – review & editing. MW: Conceptualization, Funding acquisition, Project administration, Supervision, Writing – original draft, Writing – review & editing.

## References

[B1] American Psychological Association (2022). *Integrated Primary Care Advisory Group.* Washington, DC: American Psychological Association.

[B2] Association of American Medical Colleges (2021). *Telehealth Competencies Across the Learning Continuum.* Washington, DC: Association of American Medical Colleges.

[B3] Attride-Stirling J. (2001). Thematic networks: An analytic tool for qualitative research. *Qual. Res.* 1 385–405. 10.1177/146879410100100

[B4] Blount F. Miller B. (2009). Addressing the workforce crisis in integrated primary care. *J. Clin. Psychol. Med. Settings* 16 113–119. 10.1007/s10880-008-9142-7 19148709

[B5] Bluestein D. Cubic B. (2009). Psychologists and primary care physicians: A training model for creating collaborative relationships. *J. Clin. Psychol. Med. Settings* 16 101–112. 10.1007/s10880-009-9156-9 19259793

[B6] Boon H. Mior S. Barnsley J. Ashbury F. Haig R. (2009). The difference between integration and collaboration in patient care: Results from key Informant interviews working in multiprofessional health care teams. *J. Manipulative Physiol. Ther.* 32 715–722. 10.1016/j.jmpt.2009.10.005 20004798

[B7] CCHP (2023). *What is Telehealth?* Available online at: https://www.cchpca.org/what-is-telehealth/ (accessed November 14, 2023).

[B8] Cubic B. Mance J. Turgesen J. Lamanna J. (2012). Interprofessional education: Preparing psychologists for success in integrated primary care. *J. Clin. Psychol. Med. Settings* 19 84–92. 10.1007/s10880-011-9291-y 22481240

[B9] Funk M. Saraceno B. Drew N. Faydi E. (2008). Integrating mental health into primary healthcare. *Mental Health Fam. Med.* 5 5–8. PMC2777555 22477840

[B10] Hall J. Cohen D. J Davis M. Gunn R. Blount A. Pollack D. A (2015). Preparing the workforce for behavioral health and primary care integration. *J. Am. Board Fam. Med.* 28 S41–S51. 10.3122/jabfm.2015.S1.150054 26359471 PMC7324072

[B11] Health Resources & Services Administration (2020). *Behavioral Health Workforce Education and Training (BHWET) Program for Professionals Notice of Funding Opportunity.* Rockville, MD: Bureau of Health Workforce.

[B12] Hertlein K. M Drude K. P Hilty D. M Maheu M. M (2021). Interprofessional telebehavioral health competencies framework: Implications for telepsychology. *Prof. Psychol. Res. Pr.* 52 439–448. 10.1037/pro0000400

[B13] Kinman C. Gilchrist E. Payne-Murphy J. Miller B. (2015). *Provider- and Practice-Level Competencies for Integrated Behavioral Health in Primary Care: A Literature Review.* Rockville, MD: Agency for Healthcare Research and Quality, 1–26.

[B14] Langarizadeh M. Tabatabaei M. Tavakol K. Naghipour M. Rostami A. Moghbeli F. (2017). Telemental health care, an effective alternative to conventional mental care: A systematic review. *Acta Inform. Med.* 25 240–246. 10.5455/aim.2017.25.240-246 29284913 PMC5723163

[B15] Lin T. Heckman T. Anderson T. (2022). The efficacy of synchronous teletherapy versus in-person therapy: A meta-analysis of randomized clinical trials. *Clin. Psychol. Sci. Pr.* 29 167–178. 10.1037/cps0000056

[B16] Lincoln Y. Guba E. (1985). *Naturalistic Inquiry.* London: Sage.

[B17] McCord C. Saenz J. Armstrong T. Elliott T. (2015). Training the next generation of counseling psychologists in the practice of telepsychology. *Couns. Psychol. Q.* 28 324–344. 10.1080/09515070.2015.1053433

[B18] McDaniel S. H. Grus C. L. Cubic B. A. Hunter C. L. Kearney L. K. Schuman C. C. (2014). Competencies for psychology practice in primary care. *Am. Psychol.* 69 409–429. 10.1037/a0036072 24820690

[B19] O’Donohue W. Cummings N. Cummings J. (2009). The unmet educational agenda in integrated care. *J. Clin. Psychol. Med. Settings* 16 94–100. 10.1007/s10880-008-9138-3 19112610

[B20] Papanagnou D. Sicks S. Hollander J. (2015). Training the next generation of care providers: Focus on telehealth. *Healthcare Transf.* 1 52–63. 10.1089/heat.2015.29001-psh

[B21] Perle J. (2021). Training psychology students for telehealth: A model for doctoral-level education. *J. Technol. Behav. Sci.* 6 456–459. 10.1007/s41347-021-00212-8 PMC8972773 35382354

[B22] SAMHSA-HRSA Center for Integrated Health Solutions (2019). *Standard Framework for Levels of Integrated Healthcare.* Available online at: https://www.hrsa.gov/behavioral-health/standard-framework-levels-integrated-healthcare (accessed Novemver 15, 2023).

[B23] Segal M. Giuffrida P. Possanza L. Bucciferro D. (2022). The critical role of health information technology in the safe integration of behavioral health and primary care to improve patient care. *J. Behav. Health Serv. Res.* 49 221–230. 10.1007/s11414-021-09774-0 34668115 PMC8525847

[B24] Shah B. Allen J. Chaudhury H. O’Shaughnessy J. Tyrrell C. (2022). The role of digital health in the future of integrated care. *Clin. Integr. Care* 15:100131. 10.1016/j.intcar.2022.100131

[B25] Telehealth Training (2023). *General medical education: Council on Medical Education Reports & Issue Briefs.* Available online at: https://www.ama-assn.org/topics/telehealth-training (accessed November 15, 2023).

[B26] Traube D. Cederbaum J. Taylor A. Naish L. Rau A. (2021). Telehealth training and provider experience of delivering behavioral health services. *J. Behav. Health Serv. Res.* 48 93–102. 10.1007/s11414-020-09718-0 32596804

